# Supplementary material for: Ecological interactions between marine RNA viruses and planktonic copepods
Source: Commun Biol. 2024 Nov 19;7:1507. doi: 10.1038/s42003-024-07189-z (PMC11577009; doi:10.1038/s42003-024-07189-z)
Supplement: Supplementary file 2 — Supplementary Information [file 42003_2024_7189_MOESM2_ESM.pdf]

Supplementary Figures

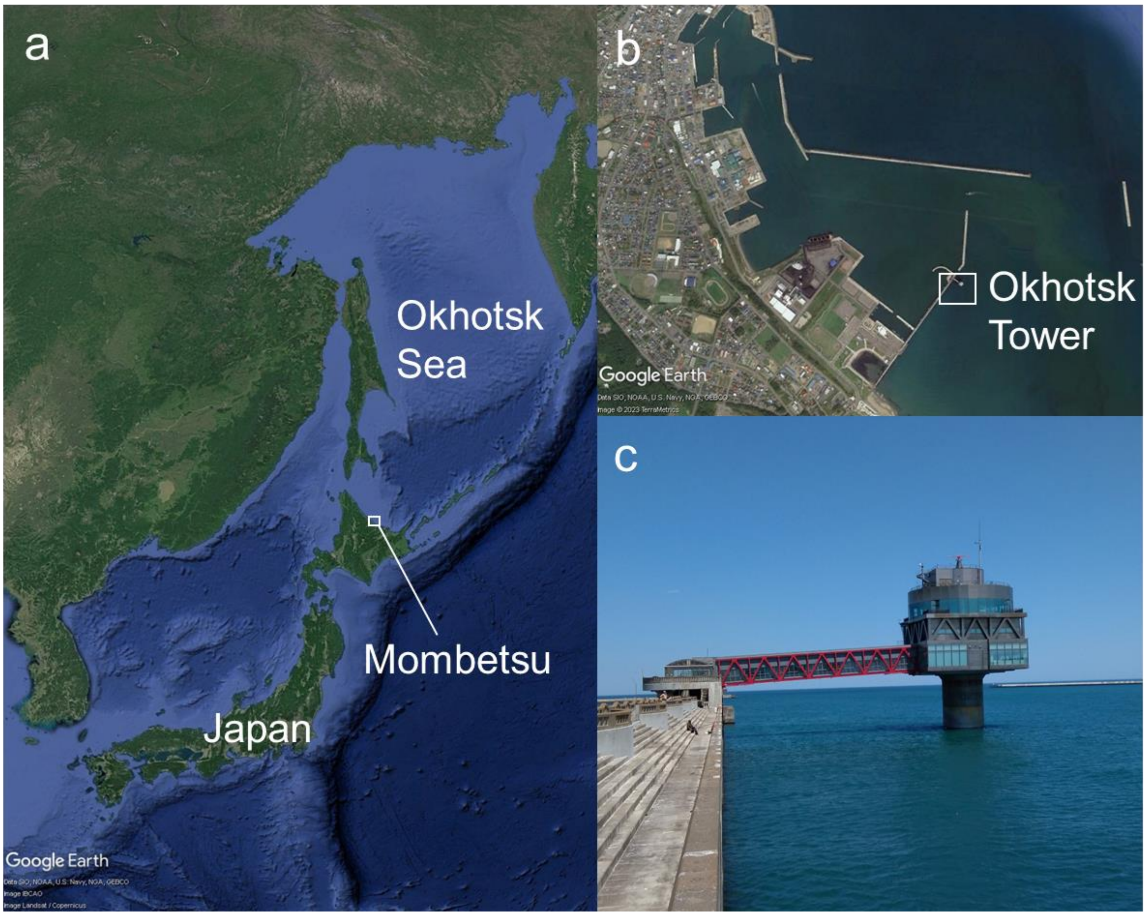

Supplementary Figure 1. Sampling location in this study. Sampling station is located in the coastal water of the southwestern Okhotsk Sea off Mombetsu (a). The Okhotsk Tower is located 1 km off the coast of Mombetsu (b and c). Satellite images were retrieved from Google Earth.

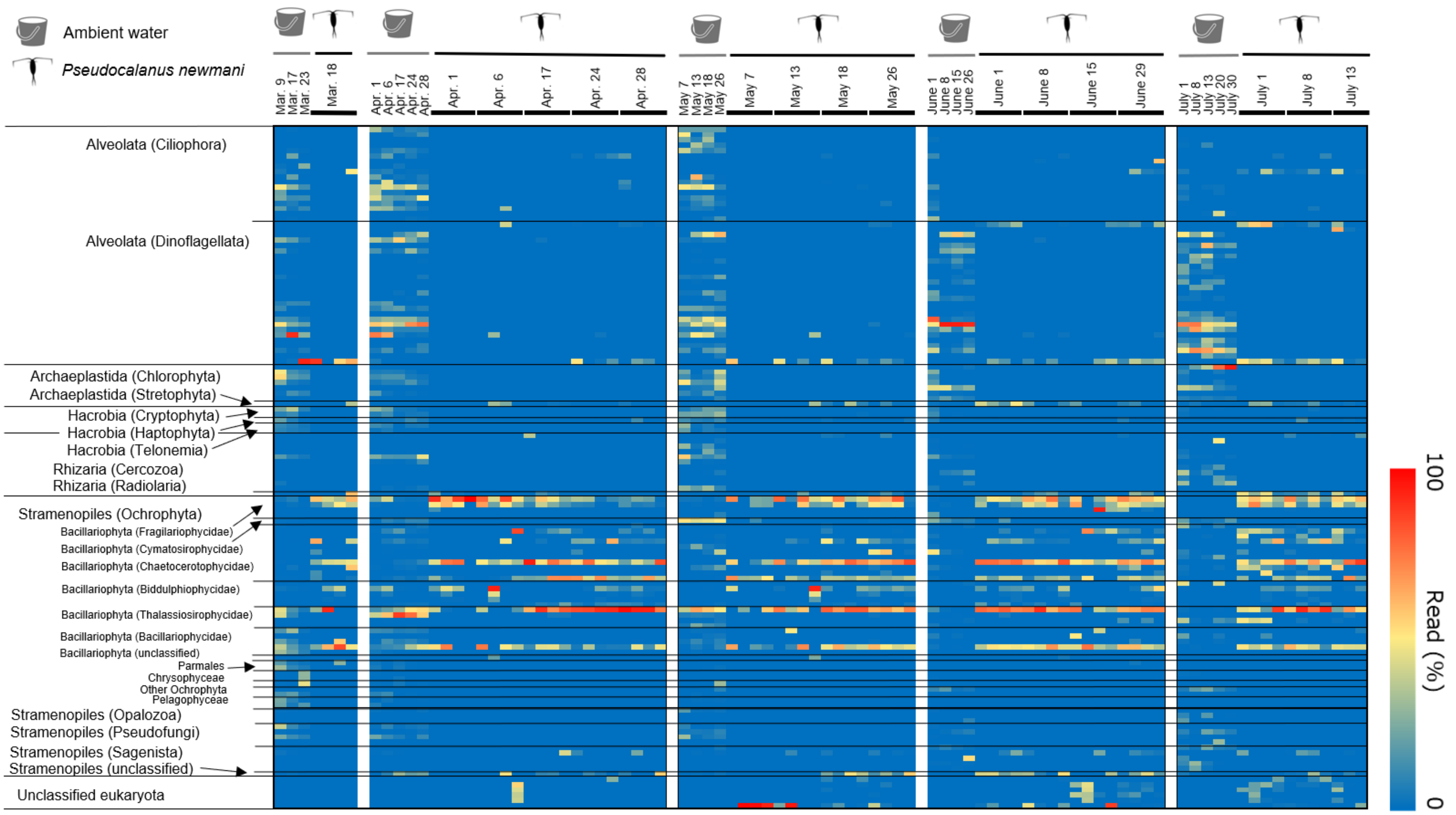

Supplementary Figure 2. OTU-level protistan communities obtained from copepods *Pseudocalanus newmani* and the ambient waters during the sampling period. Heatmaps indicate proportions of sequence reads (log-transformed).

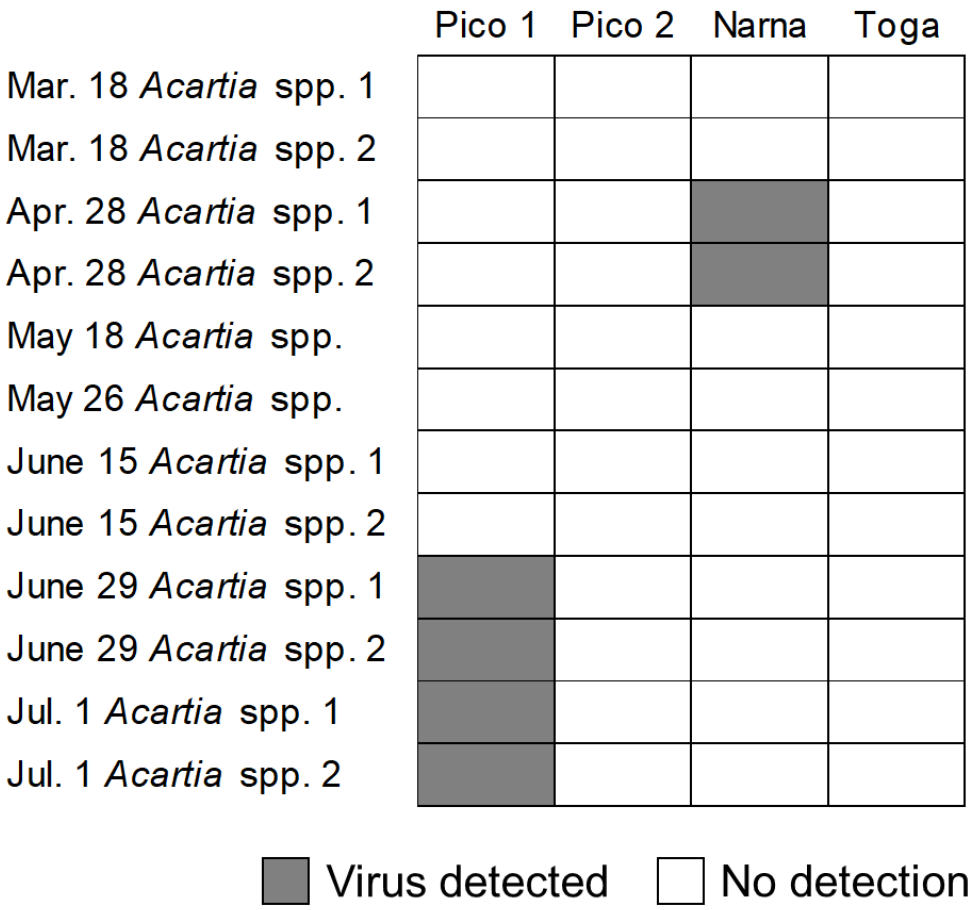

Supplementary Figure 3. Results of RT-qPCR in *Acartia* spp. for the major viruses detected from *Pseudocalanus newmani*. In each sample, five individuals of *Acartia* spp. are pooled for RNA extraction and cDNA synthesis.

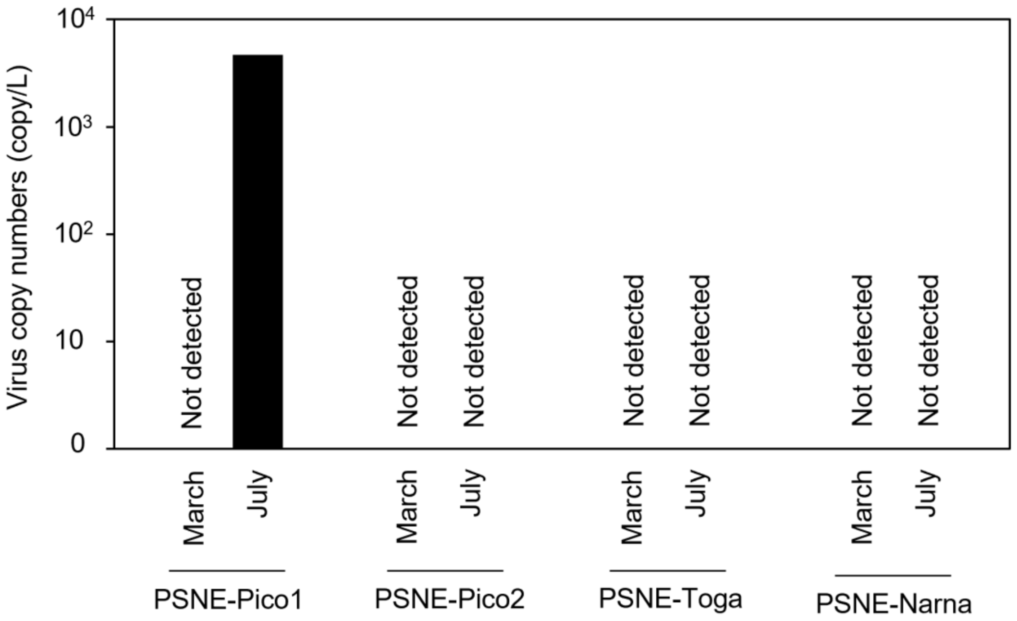

Supplementary Figure 4. RT-qPCR for detecting RNA viruses in seawaters. Sea surface waters were collected on July 20 in 2021 (July) and on March 2 in 2024 (March). 1 L of seawaters were filtered on filters with 10 µm pore size. RT-qPCR was conducted for PSNE-Pico1, PSNE-Pico2, PSNE-Toga, and PSNE-Narna.

Supplementary Tables

Supplementary Table 1. Viral contigs detected from the transcriptome data (rRNA depletion) of *Pseudocalanus newmani*. Results of BLASTX hits to known viruses are listed for each contig.

Contig	length (bp)	counts	Identity (%)	evalue	BLASTX best hit	Major viruses
TRINITY_DN135962_c0_g1_i4	9,182	914	59.6	0	YP_009505620.1 replicase polyprotein [Chaetoceros tenuissimus RNA virus 01]	PSNE-Pico1
TRINITY_DN373508_c0_g1_i1	3,571	103	32.5	7.41E-59	UHS72021.1 MAG: hypothetical protein, partial [Tombusviridae sp.]	
TRINITY_DN179973_c0_g1_i1	7,676	285	50.8	0.00E+00	QYV43051.1 MAG: polyprotein [Picornavirales sp.]	PSNE-Pico2
TRINITY_DN123894_c0_g1_i1	1,934	1271	93.2	4.71E-145	YP_009163929.1 putative replication initiation protein [Primnoa pacifica coral associated circular virus]	
TRINITY_DN123894_c0_g1_i2	2,506	953	93.2	4.78E-143	YP_009163929.1 putative replication initiation protein [Primnoa pacifica coral associated circular virus]	
TRINITY_DN68064_c0_g1_i1	3,221	3108	27.9	2.08E-43	APG77107.1 RNA-dependent RNA polymerase, partial [Changjiang narna-like virus 5]	PSNE-Narna
TRINITY_DN18295_c0_g1_i1	11,039	30789	28.4	1.44E-103	YP_009337236.1 hypothetical protein [Wenling toga-like virus]	PSNE-Toga

Supplementary Table 2. Summary of the copepod samples used for gene expression analysis. The presence of each virus is based the results using RT-qPCR (Figure 2). Two samples indicated by asterisks are excluded as outliers in the detailed analysis of gene expression.

					Presence of virus				Sequence reads			
	Date	WT (ºC)	Salinity	ID	Pico1	Pico2	Toga	Narna	Raw	Trimmomatic	SortMeRNA	Mapping (%)
1	Apr. 1	0.67	32.28	0401_2	×	×	×	×	30253387	20538116	20261463	66.96
2				0401_3	×	×	×	×	28168341	19098335	18496895	70.55
3				0401_5	×	×	×	×	30566426	21042784	18348908	70.72
4	Apr. 6	3.57	32.86	0406_3	×	×	×	×	25368721	18087088	17387881	67.96
5	Apr. 17	3.01	32.37	0417_1	×	○	×	×	24921319	16768047	15428859	72.53
6				0417_3	×	○	×	×	28110584	19745174	19348060	72.06
7	Apr. 24	4.49	33.06	0424_1	×	○	×	×	32764475	29495631	28989984	75.86
8				0424_2	×	×	○	×	31425496	27866774	27227124	76.02
9	Apr. 28	5.04	33.07	0428_1	×	×	×	○	33490251	30601244	29616547	71.54
10				0428_2	×	×	×	○	36242711	32623814	31879841	72.11
11				0428_5	×	×	×	○	31828359	27745118	27330806	73.61
12*	May 7	7.96	32.38	0507_3	×	○	×	×	41457858	36344114	35947178	77.9
13				0507_5	×	○	×	×	38852310	34118044	33317697	67.4
14				0507_6	×	×	×	×	31789966	28445028	27841148	65.79
15				0507_8	×	○	○	×	33498125	29088889	28663379	71.67
16				0507_9	×	×	×	×	34806057	30305966	29947263	74.5
17				0507_10	×	×	×	×	44779003	38991089	37209444	55.18
18	May 18	9.34	33.06	0518_1	×	×	×	×	34569710	30354572	29566265	73.42
19				0518_7	×	○	×	×	33671526	29824144	29447874	72.82
20	May 26	9.86	33	0526_1	×	×	×	×	35576929	31197591	30841047	70.13
21				0526_5	×	○	×	×	40370315	35904160	35291802	72.8
22	Jun. 1	12.03	33.4	0601_1	×	×	×	×	44345515	39095057	38410953	69.33
23				0601_9	×	○	×	×	38695995	33575723	33207803	75.89
24	Jun. 15	13.52	33.69	0615_3	×	×	×	×	27706806	20173752	19522586	72.87
25*				0615_4	×	×	×	×	26160849	18504677	17567962	69.07
26				0615_5	×	×	×	×	32715127	22485936	21993718	69.15
27	Jun. 29	13.39	33.45	0629_7	○	○	×	×	30220417	21411406	20794665	71.77
28				0629_8	○	×	×	×	29147453	19315807	18546102	68.13
29				0629_10	○	×	×	×	31623626	21862310	21059210	70

Supplementary Table 3. The Gene Ontology (GO) enrichment analysis of differentially expressed genes. The top ten significantly enriched GO terms based on the adjusted P-value for *Pseudocalanus newmani* under high viral loads are listed for upregulated and downregulated genes. CC: cellular component, BP: biological process, MF: molecular function.

	GO Term	Category	GO Name	Adj. P-value
Up-regulated	GO:0005840	CC	ribosome	5.06E-25
	GO:0003735	MF	structural constituent of ribosome	2.19E-24
	GO:1901566	BP	organonitrogen compound biosynthetic process	1.15E-23
	GO:0044237	BP	cellular metabolic process	1.70E-23
	GO:0006412	BP	translation	2.62E-23
	GO:0005198	MF	structural molecule activity	2.62E-23
	GO:0110165	CC	cellular anatomical entity	5.11E-23
	GO:0043043	BP	peptide biosynthetic process	5.11E-23
	GO:0008152	BP	metabolic process	7.21E-23
	GO:0006518	BP	peptide metabolic process	9.99E-23
Down-regulated	GO:0005622	CC	intracellular anatomical structure	1.78E-112
	GO:0005737	CC	cytoplasm	2.59E-103
	GO:0043226	CC	organelle	1.00E-95
	GO:0043229	CC	intracellular organelle	1.68E-91
	GO:0044237	BP	cellular metabolic process	6.54E-86
	GO:0008152	BP	metabolic process	2.39E-85
	GO:0071704	BP	organic substance metabolic process	2.48E-77
	GO:0110165	CC	cellular anatomical entity	1.27E-73
	GO:0009987	BP	cellular process	1.97E-73
	GO:0005739	CC	mitochondrion	8.81E-72

Supplementary Table 4. Monthly changes in genetic diversity based on mtCOI in *Pseudocalanus newmani*. mtCOI sequence numbers (N), haplotype numbers, haplotype diversity (Hd), and nucleotide diversity (π) are listed for each month.

	Mar-April	May	June	July
N	48	39	38	18
haplotype	22	24	25	11
Hd ± SD	0.90 ± 0.3	0.94 ± 0.03	0.94 ± 0.03	0.94 ± 0.03
π ± SD	0.0099 ± 0.0010	0.0123 ± 0.0005	0.01172 ± 0.0013	0.0108 ± 0.0010

Supplementary Table 5. Pairwise Φ_{ST} distances between the months based on mtCOI in *Pseudocalanus newmani* (n = 18–48). Note that no significant differences were observed between any months (α = 0.008 after Bonferroni correction).

		1	2	3	4
1	March-April				
2	May	0.032			
3	June	0	0.034		
4	July	0	0	0	

Supplementary Table 6. Primers and probes used in this study.

Target gene	Primer, probe, and PNA	Sequence
COI	LCO1490_Pseudocalanus	5'-GRTCRTGTAAYCATAAAGATATYGG-3'
	HCO2198_Pseudocalanus	5'-TAYACYTCAGGGTGHCCAAARAAYCA-3'
PSNE-Pico1	Pico1_1894F	5'-CGAGTGACAGGTTCGAAAGA-3'
	Pico1_2032R	5'-GAGCGACTTCATGTTCTGGA-3'
	Pico1_1979P	5'-[FAM]TCGGTCAAGTTTTGGAATTGTTTTCACA[TAMRA]-3'
PSNE-Pico2	Pico2_3F	5'-AAGTGGTTCTGGGAATCAGG-3'
	Pico2_3R	5'-TGAGGCAAGCCTTGTAGATG-3'
	Pico2_3p	5'-[FAM]ACGTGGACCAGTTCGACATCAAGGT[TAMRA]-3'
PSNE-Narna	Narna5F	5'-CACTGCGGAGCTTACCATTA-3'
	Narna5R	5'-AGGGTCTCCCGAGGAATACT-3'
	Narna5p	5'-[FAM]ATCGTTCTCCTTGGCCATATCCACC[TAMRA]-3'
PSNE-Toga	Toga2F	5'-ATGCAATTCTTCCTCCCAAC-3'
	Toga2R	5'-GGACGGGTTACAGCTCAAAT-3'
	Toga2p	5'-[FAM]CTAACGTCGGATCACAATGCCGC[TAMRA]-3'
18S V7-V9	18S-V7F	5'-TGGAGYGATHTGTCTGGTTDATTCCG-3'
	18S-V9R	5'-TCACCTACGGAWACCTTGTTACG-3'
	PSNE-V9-PNA	5'-GATCACTCTGCCTGCCT-3'
metabarcoding 1st	18S-V7F with adaptor	5´-ACACTCTTTCCCTACACGACGCTCTTCCGATCT+[18S-V7F]-3´
	18S-V9R with adaptor	5´-GTGACTGGAGTTCAGACGTGTGCTCTTCCGATCT+[18S-V9R]-3´
metabarcoding 2nd	forward	5´-AATGATACGGCGACCACCGAGATCTACAC+[8 bp index]+ACACTCTTTCCCTACACGACGC-[illegible]
	reversed	5´-CAAGCAGAAGACGGCATACGAGAT+[8 bp index]+GTGACTGGAGTTCAGACGTGTG-3´
