# Supplementary material for: Ecological interactions between marine RNA viruses and planktonic copepods
Source: Commun Biol. 2024 Nov 19;7:1507. doi: 10.1038/s42003-024-07189-z (PMC11577009; doi:10.1038/s42003-024-07189-z)
Supplement: Supplementary file 3 — Reporting Summary [file 42003_2024_7189_MOESM3_ESM.pdf]

nature portfolio

Corresponding author(s): Junya Hirai

Last updated by author(s): Oct 31, 2024

Reporting Summary

Nature Portfolio wishes to improve the reproducibility of the work that we publish. This form provides structure for consistency and transparency in reporting. For further information on Nature Portfolio policies, see our Editorial Policies and the Editorial Policy Checklist.

Statistics

For all statistical analyses, confirm that the following items are present in the figure legend, table legend, main text, or Methods section.

n/a	Confirmed	
☐	☒	The exact sample size (*n*) for each experimental group/condition, given as a discrete number and unit of measurement
☐	☒	A statement on whether measurements were taken from distinct samples or whether the same sample was measured repeatedly
☐	☒	The statistical test(s) used AND whether they are one- or two-sided<br>*Only common tests should be described solely by name; describe more complex techniques in the Methods section.*
☐	☒	A description of all covariates tested
☐	☒	A description of any assumptions or corrections, such as tests of normality and adjustment for multiple comparisons
☐	☒	A full description of the statistical parameters including central tendency (e.g. means) or other basic estimates (e.g. regression coefficient) AND variation (e.g. standard deviation) or associated estimates of uncertainty (e.g. confidence intervals)
☐	☒	For null hypothesis testing, the test statistic (e.g. *F*, *t*, *r*) with confidence intervals, effect sizes, degrees of freedom and *P* value noted<br>*Give P values as exact values whenever suitable.*
☒	☐	For Bayesian analysis, information on the choice of priors and Markov chain Monte Carlo settings
☒	☐	For hierarchical and complex designs, identification of the appropriate level for tests and full reporting of outcomes
☐	☒	Estimates of effect sizes (e.g. Cohen's *d*, Pearson's *r*), indicating how they were calculated

Our web collection on statistics for biologists contains articles on many of the points above.

Software and code

Policy information about availability of computer code

Data collection	n/a
Data analysis	Data were analyzed using Geneious (for haplotype analysis), OmicsBox, R, R-PCA, and R-GLM.

For manuscripts utilizing custom algorithms or software that are central to the research but not yet described in published literature, software must be made available to editors and reviewers. We strongly encourage code deposition in a community repository (e.g. GitHub). See the Nature Portfolio guidelines for submitting code & software for further information.

Data

Policy information about availability of data

All manuscripts must include a data availability statement. This statement should provide the following information, where applicable:

- Accession codes, unique identifiers, or web links for publicly available datasets
- A description of any restrictions on data availability
- For clinical datasets or third party data, please ensure that the statement adheres to our policy

Information are available in the section 'Data availability'.

Research involving human participants, their data, or biological material

Policy information about studies with human participants or human data. See also policy information about sex, gender (identity/presentation), and sexual orientation and race, ethnicity and racism.

Reporting on sex and gender	n/a
Reporting on race, ethnicity, or other socially relevant groupings	n/a
Population characteristics	n/a
Recruitment	n/a
Ethics oversight	n/a

Note that full information on the approval of the study protocol must also be provided in the manuscript.

Field-specific reporting

Please select the one below that is the best fit for your research. If you are not sure, read the appropriate sections before making your selection.

☐ Life sciences ☐ Behavioural & social sciences ☒ Ecological, evolutionary & environmental sciences

For a reference copy of the document with all sections, see nature.com/documents/nr-reporting-summary-flat.pdf

Ecological, evolutionary & environmental sciences study design

All studies must disclose on these points even when the disclosure is negative.

Study description	Weekly sampling of zooplankton and environmental waters in the coastal station in the Okhotsk Sea to investigate ecological interactions between marine RNA viruses and planktonic copepods
Research sample	Weekly collections of zooplankton and seawaters
Sampling strategy	Sampling was conducted to cover different season. We did not analyzed samples after disappearance of the target species of copepods. This sampling strategy is mentioned in the section 'Methods'.
Data collection	Zooplankton samples were collected using NORPAC net. Seawaters were collected using a bucket. All sampling information are recorded as a part of the monitoring program managed by Mombetsu city (Japan).
Timing and spatial scale	Samples were collected from the single monitoring station in the Okhotsk Sea off Japan to cover temporal changes of zooplankton and viruses. As described above, samples were collected weekly to cover different season from March to July. We did not analyzed samples after disappearance of the target species of copepods. This sampling strategy is mentioned in the section 'Methods'.
Data exclusions	No samples were excluded.
Reproducibility	One bulk zooplankton sample was collected in each sampling data, and 10 copepod individuals were selected from each bulk sample.
Randomization	10 female copepods were randomly selected from a bulk sample in each sampling day, and sampling days were classified in to different seasons based on environmental parameters (e.g. water temperature).
Blinding	No blinding. Binding treatments to avoid biases were not necessary in this study.

Did the study involve field work? ☒ Yes ☐ No

Field work, collection and transport

Field conditions	We carried out weekly samplings at the single monitoring station in the Okhotsk Sea off Japan to cover zooplankton samples at different seasons. Environmental parameters including temperature, salinity, chlorophyll, and nutrients are represented in the manuscript.
Location	We include information of location (Okhotsk Tower; 44° 333 20.2' N, 143° 22.9' E) and water depth (10 m) in the manuscript.
Access & import/export	One of our co-author is belonging to Mombetsu city which operate the ocean monitoring in our study site, and no permissions were

Access & import/export	required for samplings of zooplankton and seawaters. All samplings and experiments were performed in Japan, and no permissions were required for transportations of samples.
Disturbance	No disturbances were caused by a tow of small plankton net and by a water sampling.

Reporting for specific materials, systems and methods

We require information from authors about some types of materials, experimental systems and methods used in many studies. Here, indicate whether each material, system or method listed is relevant to your study. If you are not sure if a list item applies to your research, read the appropriate section before selecting a response.

Materials & experimental systems

n/a	Involved in the study
☒	☐ Antibodies
☒	☐ Eukaryotic cell lines
☒	☐ Palaeontology and archaeology
☐	☒ Animals and other organisms
☒	☐ Clinical data
☒	☐ Dual use research of concern
☒	☐ Plants

Methods

n/a	Involved in the study
☒	☐ ChIP-seq
☒	☐ Flow cytometry
☒	☐ MRI-based neuroimaging

Animals and other research organisms

Policy information about studies involving animals; ARRIVE guidelines recommended for reporting animal research, and Sex and Gender in Research

Laboratory animals	n/a
Wild animals	Bulk zooplankton samples were collected using a small plankton net (NORPAC net) and preserved in RNAlater. In the laboratory, adult female Pseudocalanus newmani (Copepoda) were picked up for DNA and RNA extractions.
Reporting on sex	We used female copepods in this study. Population dynamics of copepods are mainly carried out using female individuals, because males are rare in the ocean.
Field-collected samples	Zooplankton samples were preserved in RNAlater immediately after samplings in the field.
Ethics oversight	No ethical approvals are required to collect marine zooplankton and seawater samples.

Note that full information on the approval of the study protocol must also be provided in the manuscript.

Plants

Seed stocks	n/a
Novel plant genotypes	n/a
Authentication	n/a
